# Supplementary material for: Dispersion state phase diagram of citrate-coated metallic nanoparticles in saline solutions
Source: Nat Commun. 2020 Oct 27;11:5422. doi: 10.1038/s41467-020-19164-3 (PMC7591489; doi:10.1038/s41467-020-19164-3)
Supplement: Supplementary file 1 — Supplementary Information [file 41467_2020_19164_MOESM1_ESM.pdf]

Supplementary Information

Dispersion state phase diagram of citrate-coated metallic nanoparticles in saline solutions

Sebastian Franco-Ulloa[1], Giuseppina Tatulli[2], Sigbjørn Løland Bore[3], Mauro Moglianetti[2], Pier Paolo Pompa[2,*], Michele Cascella[3,*], and Marco De Vivo[1,*]

1. Molecular Modeling and Drug Discovery Lab, Istituto Italiano di Tecnologia, via Morego 30, 16163 Genova, Italy
2. Nanobiointeractions & Nanodiagnostics, Istituto Italiano di Tecnologia, via Morego 30, 16163 Genova, Italy.
3. Department of Chemistry and Hylleraas Centre for Quantum Molecular Sciences, University of Oslo, P.O. Box 1033 Blindern, 0315 Oslo, Norway

*Corresponding authors:

Dr. Pier Paolo Pompa – Email: pierpaolo.pompa@iit.it

Dr. Michele Cascella – Email: michele.cascella@kjemi.uio.no

Dr. Marco De Vivo – Email: marco.devivo@iit.it

Contents

1. Model development 3
a. Solvation free energy calculations – thermodynamic integration protocol 3
b. Citrate binding electronic energy 4
c. Entropic contribution to binding 5
d. Derivation of $\Delta \mathrm{Elig/lig}$ 6
e. Derivation of the polar solvation term within $\Delta \mathrm{Gsolv}(N)$ 7
f. Explicit citrate coarse-grained model for solvation free energy calculations 8
2. Estimation of citrate surface coverage 8
3. Counterion residence time analysis and sampling of slow degrees of freedom 9
4. Absorbance profiles 13
5. Correlation between well-depth ε and free energy of dimerization 13
6. Shear planes distributions 14
7. Coarse-grained models for metal NPs 14
a. The building of coarse-grained hydrophobic NPs 14
b. The building of charged NPs for the building of dispersion state phase diagrams 14
8. AuNPs TEM images 16
9. Supplementary references 16

1. Model development

As described in the main text, by minimizing the change of free energy ΔG_{cap} we recover the value of *N* of the highest likelihood. The first contribution, ΔG_{desolv}, can be expressed in terms of the desolvation energy of a single ligand (ΔG^{lig}_{desolv}) multiplied by *N* and that of the metallic NP (ΔG^{M}_{desolv}). The linear trend followed by ΔG^{lig}_{desolv} is plotted as a function of *N* in Supplementary Fig. 1. Note, moreover, that ΔG^{M}_{desolv} cancels out with the apolar contribution from the solvation energy of the capped nanoparticle ΔG^{apolar}_{solv}.

a. Solvation free energy calculations – thermodynamic integration protocol

In order to calculate the solvation free energy ΔG^{lig}_{desolv}, we adopted the thermodynamic integration methodology. In our case, we first immersed an explicit coarse-grained citrate molecule in a box of polarizable water molecules parametrized with the refPol force field.[1–3] The free energy calculation was stratified into 31 runs with increasing values of the coupling parameter, λ. The spacing between windows was of 0.05 for $\lambda < 0.6$ and 0.02 elsewhere. A value of 0.5 was used as the alpha parameter in the soft core potential.[4,5] For each window in the calculation, two minimizations were performed for a maximum of 5000 steps each. The first of them used the steepest descent method, and the second one followed the l-BFGS algorithm. Once the systems were minimized, we proceeded to thermalize and pressurize to 310K and 1 bar with a 10 ns long simulation in the NPT statistical ensemble. These employed the Berendsen thermostat (τ_T = 2.0 ps) and the isotropic Berendsen barostat (τ_P = 5.0 ps and $\kappa = 4.5 \times 10^{-4}$ bar^{-1}). After the equilibration, a production run was performed for each window for 25 ns using a time-step of 20 fs and switching the Berendsen barostat for its Parrinello-Rahman counterpart (τ_P = 14.0 ps and $\kappa = 4.5 \times 10^{-4}$ bar^{-1}).[6] The variations in the Hamiltonian and its derivative respect to λ were saved every 10 steps (0.2 ps). Finally, the solvation free energy was estimated with Bennett's acceptance ratio method.[7] The aforementioned procedure was performed twice to sequentially switch off the electrostatic and van der Waals interactions, respectively.

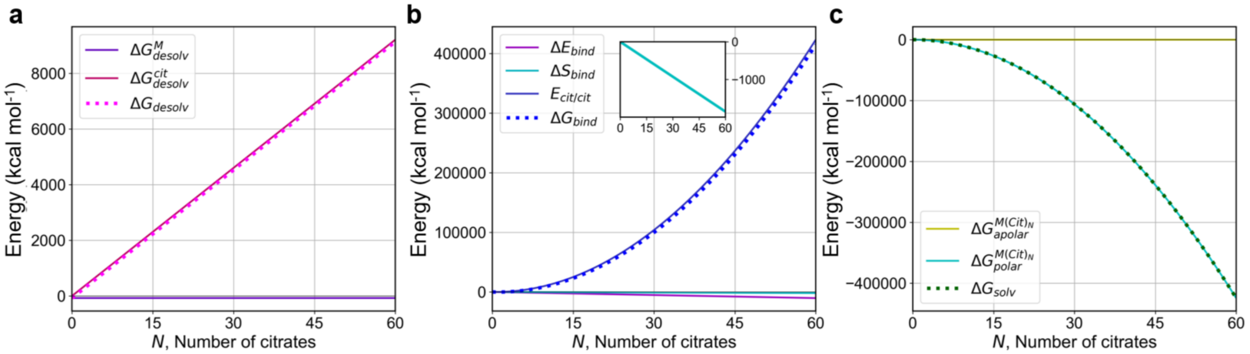

Supplementary Fig. 1 The different contributions accounted by the main terms included in the theoretical model as a function of the number of bound citrate molecules. The plots shown are built for α = 3. **a.** Breakdown of ΔG_{desolv}. **b.** Breakdown of ΔG_{bind}. **c.** Breakdown of ΔG_{solv}.

b. Citrate binding electronic energy

In order to determine the number of equivalent binding modes of citrate onto gold surfaces, we first generated a realistic, all-atom representation of a gold NP. For this, we used the NanoCrystal web server.[8] The circumradius parsed to the server was 1.5 nm, and the surface energies utilized for the gold facets were those derived by Skriver and co-workers.[9] The result was an NP formed by six (100) planes and eight (111) planes as those depicted in Supplementary Fig. 2. As described by Al-Johani et al.,[10] the binding of citrate may take place via one (mono-) or two (bidentate) carboxylate groups. The multiplicities (δ) of each type of monodentate and bidentate binding mode are shown in Supplementary Fig. 2. The displayed multiplicities consider the interchangeability of the three available carboxylate groups and that of their two oxygen atoms. Importantly, these values are expressed for individual planes, not for the entire NP. Notably, for the bidentate modes, each pose has a different associated binding energy depending on the pair of carboxylates involved. That is, if the two terminal groups are bound, the energetics resemble those of glutarate, whereas if the binding takes place with one terminal and the center carboxylate, the energetics should resemble that of succinate. Thus, with this information, we used Supplementary Equation (1) to calculate the ensemble average of the binding electronic energy for citrate onto gold in vacuum.

$$\Delta E_{bind} = \frac{\sum_i^{modes} \epsilon_i \delta_i e^{-\beta \epsilon_i}}{\sum_i^{modes} \delta_i e^{-\beta \epsilon_i}} \qquad (1)$$

Here, ϵ_i is the electronic binding energy associated with the i-th binding mode, δ_i its multiplicity, and $e^{-\beta\epsilon_i}$ the corresponding Boltzmann weighting factor. Following this scheme, we estimated the average electronic binding energy between citrate and gold to be -40.9 kcal mol^{-1}.

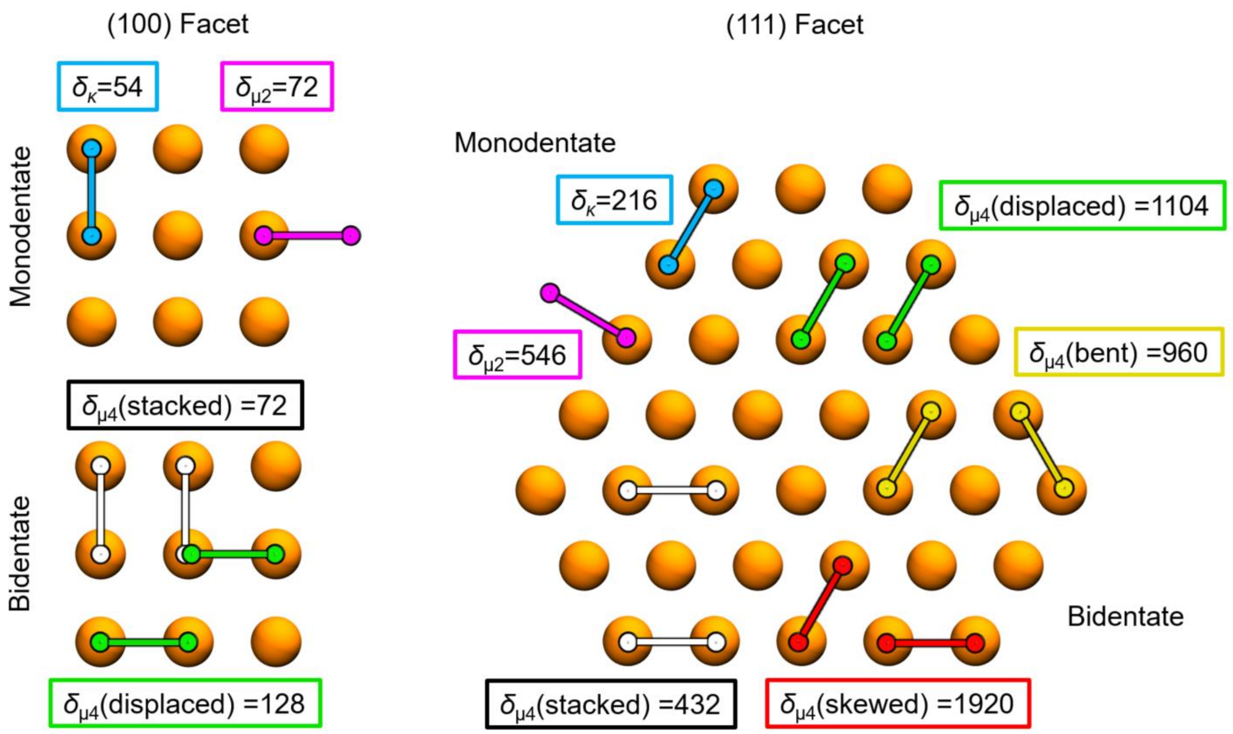

Supplementary Fig. 2 Graphical representation of all the possible binding modes for acetate, succinate, and glutarate as described by Al-Johani, et al[10] and using their same nomenclature. Each binding mode is accompanied by its multiplicity, that is, equivalent binding orientations in the grid considering the interchangeability of the carboxylate groups and their respective oxygen atoms. Orange spheres are gold atoms from a particular lattice facet.

c. Entropic contribution to binding

In order to account for the entropic contribution of binding a ligand (e.g., citrate) onto a metallic surface, three terms were considered. These terms account for the decrease in translational and rotational degrees of freedom as well as the insurgence of vibrational modes upon binding. The total change in entropy can thus be written as in Supplementary Equation (2).

$$\Delta S_{bind} = \Delta S_{trans}^{\downarrow} + \Delta S_{rot}^{\downarrow} + \Delta S_{vib}^{\uparrow} \qquad (2)$$

The translational loss of entropy is calculated from the entropy of an ideal gas employing the Sackur-Tetrode equation (Supplementary Equation (3)).

$$\Delta S_{\mathrm{trans}}^{\downarrow} = R\left[\ln\left(\frac{10^{-3}}{cN_A\Lambda^3}\right) + \frac{5}{2}\right] \qquad (3)$$

Where c is the molar concentration of citrate, R is the ideal gas constant, N_A is Avogadro's number, and Λ is the thermal de Broglie wavelength given by Supplementary Equation (4):

$$\Lambda = \left(\frac{2\pi\hbar^2}{mkT}\right)^{\frac{1}{2}} \qquad (4)$$

Where m is the mass of one ligand molecule, k is Boltzmann's constant, and T the temperature which in this case was set to 310K. Similarly, the loss of molecular rotational entropy was calculated from Supplementary Equation (5).

$$\Delta S_{rot}^{\downarrow} = 3R\left[\frac{1}{2} + \ln\left(\frac{2I_g kT}{\hbar^2}\right)\right] \qquad (5)$$

Where I_g is the geometric mean of the molecule's moments of inertia, as stated by Supplementary Equation (6).

$$I_g = \sqrt[3]{I_1 I_2 I_3} \qquad (6)$$

Where I_1, I_2, and I_3 are the three principal moments of inertia calculated from the diagonalization of the tensor of inertia. In our case, we derived the moments of inertia from an all-atom molecular dynamics simulation of a single citrate molecule immersed in water. The setup of this simulation is described in Supplementary Discussion 1f. Finally, the decrease in vibrational entropy arises from the formation of metal-ligand bonds. Their contribution was estimated from Supplementary Equation (7).

$$\Delta S_{\mathrm{vib}}^{\uparrow} = R\left[\frac{1}{2} - \ln\frac{kT}{\hbar\omega}\right] \; (7)$$

Where ω is the stretching frequency of the formed bond. For the specific case of citrate, the frequency for the Au-O bonds was taken as 1638 cm^{-1}, as measured by Wulandari et al.[11] It is noteworthy that, as evidenced in Supplementary Fig. 1, the entropic correction for citrate binding onto gold is negligible compared to the process's enthalpy.

d. Derivation of $\Delta E_{lig/lig}$

The electric potential energy of a charge density distribution $\rho(\mathbf{r})$ is given by Supplementary Equation (8).

$$U(\mathbf{r}) = \frac{1}{2}\int v(\mathbf{r})\rho(\mathbf{r})\mathrm{dV} \qquad (8)$$

In which $v(\mathbf{r})$ is the electric potential generated by the charge distribution, $\rho(\mathbf{r})$, and the integral covers the entire space. For the case of a hollow sphere with uniform surface charge density, the charge distribution function can be written as in Supplementary Equation (9).

$$\rho(N,\mathbf{r}) = \frac{Q(N)}{4\pi r^2}\delta(r - \mathrm{R_{NP}}) \quad (9)$$

Where $\mathrm{R_{NP}}$ is the radius of the NP, $Q(N)$ is the total charge, and δ is the Dirac delta function. In order to generalize our model, we consider a generic, polyprotic ligand that adopts a mean deprotonation state α when bound to the NP. For a ligand with three acid protons like citrate, the mean deprotonation state lies between 0 and 3, that is $\alpha \subseteq [0,3]$. Then, the total charge may be written as in Supplementary Equation (10).

$$Q(N) = -\alpha N \quad (10)$$

On the other hand, plugging (9) into Gauss's Law, it is possible to obtain the electric potential generated by the charge distribution in a medium of dielectric constant ϵ_r. This is given by Supplementary Equation (11).

$$v(N,\mathbf{r}) = \begin{cases} 0, & r < \mathrm{R_{NP}} \\ -\frac{\alpha N}{4\pi\epsilon_0\epsilon_r}, & r \geq \mathrm{R_{NP}} \end{cases} \quad (11)$$

Substituting (9) and (11) into (8), and solving the integral, we obtain an expression for the electric potential energy as a function of the number of bound ligands (Supplementary Equation (12)).

$$U(N,\epsilon_r) = \frac{\alpha^2 N^2}{8\pi\epsilon_0\epsilon_r \mathrm{R_{NP}}} \quad (12)$$

Note that this expression assumes each incoming charge as independent from the rest; however, each ligand constraints the incorporation of α fundamental charges to the system. Thus, we can write $\Delta\mathrm{E_{lig/lig}}$ as in Supplementary Equations (13) and (14).

$$\Delta\mathrm{E_{lig/lig}}(N) = U(N,\epsilon_r = 1) - N * U(N = 1,\epsilon_r = 1) \quad (13)$$

$$\Delta\mathrm{E_{lig/lig}}(N) = \frac{\alpha^2 N}{8\pi\epsilon_0 \mathrm{R_{NP}}}(N-1) \quad (14)$$

e. Derivation of the polar solvation term within $\Delta\mathbf{G_{solv}}(\boldsymbol{N})$

The polar component for the solvation of a sphere with a charge distribution given by $\rho(N,\mathbf{r})$ can be written as in Supplementary Equation (15).

$$\Delta\mathrm{G_{solv}^{polar}}(N) = U(N,\epsilon_r = \epsilon_{\mathrm{H_2O}}) - U(N,\epsilon_r = 1) \quad (15)$$

By substituting (12) into (15), we obtain (16).

$$\Delta\mathrm{G_{solv}}(N) = \frac{\alpha^2 N^2}{8\pi\epsilon_0 \mathrm{R_{NP}}}\left(\frac{1}{\epsilon_{\mathrm{H_2O}}} - 1\right) \quad (16)$$

Supplementary Equations (14) and (16) imply that the citrate-citrate and citrate-solvent interactions is mainly electrostatic. Thus, the theoretical framework presented here remains valid for small, charged ligands that do not affect greatly the NP's hydrophobicity.

f. Explicit citrate coarse-grained model for solvation free energy calculations

For the calculation of the solvation free energy of one citrate molecule (i.e., ΔG_{desolv}^{lig} in Supplementary Discussion 1a), we adopted an explicit coarse-grained representation. The model for citrate corresponded to three consecutive beads of type Qda as implemented in the standard Martini force field. Each bead had a charge of -1 to reproduce the most populated protonation state of fully solvated citrate molecules. The parameters used for the chemical bonds were k_b = 5000 kJ mol^{-1} nm^{-2} and x_0 = 0.52 nm, the typical inter-bead distance in the Martini force field. The parameters used for the angle between the beads were k_θ = 5000 kJ mol^{-1} rad^{-2} and θ_0 = 111.11°.

The explicit-citrate coarse-grained model was parametrized against atomistic MD simulation. In detail, the equilibrium angle θ_0 was obtained from a 100 ns-long, all-atom MD simulations of citrate (parametrized with the GAFF force field[13]) in TIP3P water.[16] This simulation was also used to calculate the three main moments of inertia for a citrate molecule (i.e., I_i in Supplementary Discussion 1c). In addition, the same atomistic simulation was used to obtain the projected area per citrate molecule onto our AuNPs (i.e., a in Supplementary Discussion 2).

2. Estimation of citrate surface coverage

The fraction of the surface area covered by citrate molecules, φ, was calculated by means of Supplementary Equation (17).

$$\varphi(N) = \frac{Na}{A} \qquad (17)$$

In which A is the total surface area of the NP, N is the number of citrate molecules bound to the metallic core, and a is the area covered by one citrate molecule. The total area, A, was estimated as $4\pi r \mathrm{R}_{\mathrm{NP}}^2$ yielding a value of c.a. 21.2 nm^2. The number N was estimated to be between 28 and 33 from the model described in the Results and Discussion section from the main text and deepened in Supplementary Discussion 1. The area per citrate molecule, a, was calculated from the all-atom MD simulations of citrate in water described in Supplementary Discussion 1f. This calculation was performed by projecting the van der Waals surface of one citrate molecule onto the XY plane

for a series of MD-accessed conformers and calculating the projected area. Specifically, the structure of citrate was withdrawn every 10 ps from the trajectory file. For each of these states, 100 random rotations were generated. Then, the molecular surface of each of these configurations was projected onto the XY plane, and the area was estimated. The distribution of all the calculated areas is shown in Supplementary Fig. 3.

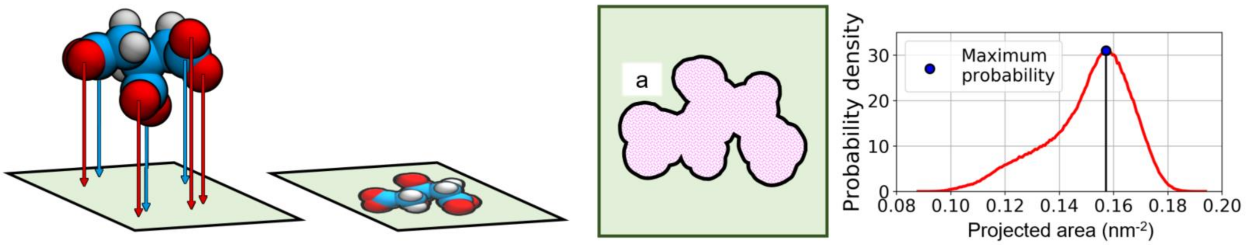

Supplementary Fig. 3 Illustration of the method employed for the calculation of the projected surface area of citrate. For every frame in the trajectory, a series of orientations were sampled. The van der Waals surface was then projected onto the XY plane, and its area was calculated. The figure also shows the probability distribution function of the projected area, which has a maximal probability at 0.16 nm^2. Carbon atoms are shown in cyan, oxygen atoms in red, and hydrogen atoms in white. The XY plane is colored green.

3. Counterion residence time analysis and sampling of slow degrees of freedom

For this study, we had to ensure that the rotational degrees of freedom of the implicit-citrate coarse-grained NPs were exhaustively sampled at short values of CV1. To understand the effectiveness of our PMF calculations, we evaluated four distinct sampling protocols. The first protocol (referred to as P1) consisted of equal, short production runs. In P1, all the windows were simulated for 25 ns, each. In the second protocol, P2, the setup was similar. The frames where CV1 > 1.2 nm were simulated as in P1, but the windows with CV1 ≤ 1.2 nm were simulated for 250 ns. P3, the third protocol, implied more brusque modifications onto the coordinate files of frames with CV1 ≤ 1.2 nm. From these frames on P2, the counterions residing 0.7 nm from any metallic bead for at least 50 ns were exchanged with water molecules in bulk. Once the ions were displaced, the same frames were simulated for 250 ns. The last protocol, P4, included the simulated annealing described in the Methods section of the main text. The described protocols were tested on two systems. Both systems involved NPs with a total charge of -40 e (charge density 1.9 e nm^{-2}), and they had respective ionic strengths of 30 mM and 70 mM.

As can be seen from Supplementary Fig. 4a, the estimated free energy of dimerization varies significantly depending on the assumed sampling protocol. It is important to note that, even though the charged beads follow a uniform distribution on the NPs, their placement is not perfectly symmetrical. This leads to the distinguishability of states depending on the relative orientation of both NPs. When P1 and P2 are contrasted, it becomes evident that the energy profiles are smoothened as more time was simulated in each window. This indicates that the NP explored more widely their rotational degrees of freedom. The local energy minima visited by the particles is a consequence of the formation of a suboptimal network of salt bridges between metallic, charged beads, and sodium counterions at different NP orientations.

To understand better the dynamics of the surrounding ions, we computed the residence time of each binding event onto the metallic surfaces. Supplementary Fig. 4b shows the residence time for P2 (i.e., sampling for 250 ns when CV1 $\leq$ 1.2 nm) in the two test cases, that is, an NP with total charge -40 e (charge density 1.9 e nm^{-2}) at an ionic strength of 30 and 70 mM. Even though most of the binding events persisted for less than 50 ns, there was a non-negligible amount of ions permanently bound to the particles. In order to ensure that these ions were not kept in their position by steric traps, we calculated the same residence times as a function of the inter-particle distance (CV1, Supplementary Fig. 4c). Interestingly, as the NPs came closer together, the number of high-residence binding events increased. This trend suggests that the binding of the ions was mediated by electrostatic interactions, which in turn were enhanced as the two rigid bodies approached each other.

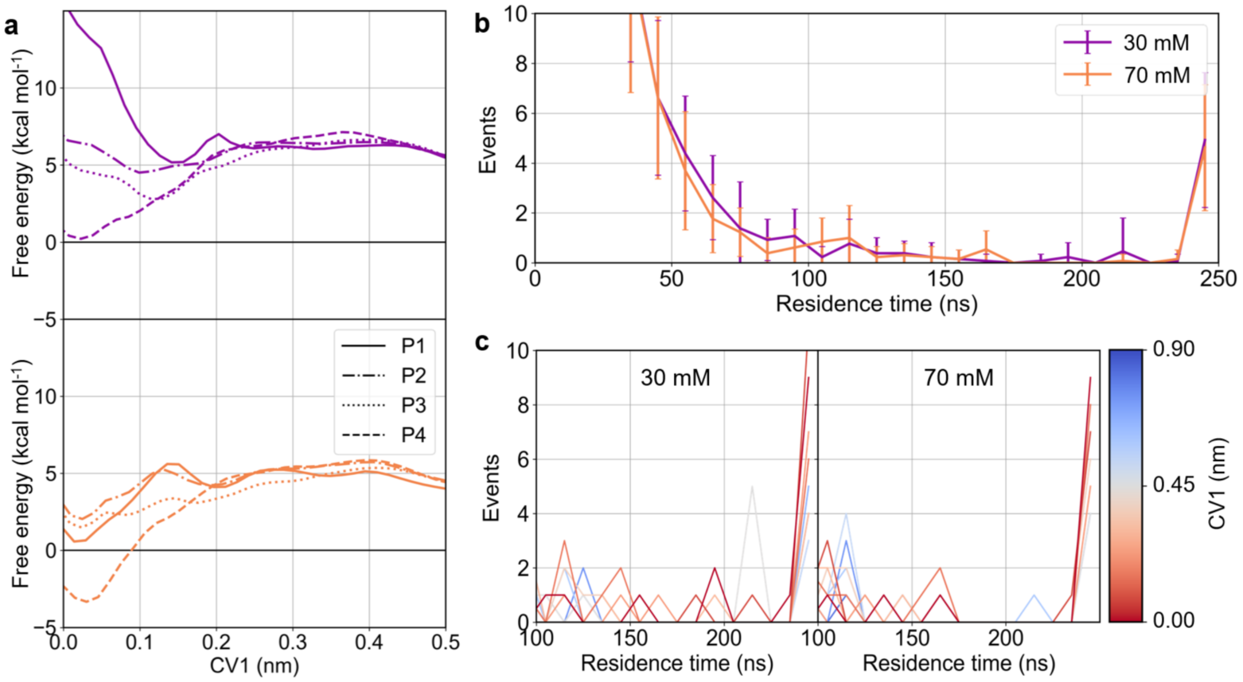

Supplementary Fig. 4 Assessment of the best sampling protocol. **a.** PMF profiles obtained for NPs with a net charge of -40 e (charge density 1.9 e nm^{-2}) in a solution of ionic strength 30 mM (purple) and 70 mM (orange). **b.** Residence times of sodium ions binding onto the surface of the NPs for the protocol P2. The solid lines show the average and standard deviation calculated from the windows with CV1 $\leq$ 1.2 nm. **c.** The residence time of sodium ions as a function of the inter-particle distance (CV1) for protocol P2.

Based on these results, we adopted two approaches that could enhance the sampling of the diffusion of the sodium counterions. The first of these approaches, P3, consisted of the manual replacement of the persistent ions with bulk water molecules. Upon this replacement, a new set of ions appeared in the same halo-like structure (Fig. 3). Since the relative position of the hydrophobic NPs and the respective salt bridges differ from P1 and P2, the free energy profiles varied slightly (Supplementary Fig. 4a). Nonetheless, P3 introduced an abrupt alteration to the free energy landscape leading to an instantaneous push of the system away from its thermodynamic equilibrium. In light of these observations, a final strategy, P4, was considered. In the protocol P4, the conformational space of the counterions was extensively sampled by performing a simulated annealing prior to the data collection run. As can be seen from the two test cases exhibited in Supplementary Fig. 4a, the proper phase space sampling leads to a significant decrease in the free energy of aggregation. P4 was the chosen sampling protocol for the simulations discussed in the main text of this work.

It is important to note that the sodium ions' residence times computed from our coarse-grained models offer a qualitative description of the lability of the counterions present, rather than structural insights on their exact binding mode onto metallic NPs. Thus, in order to verify the results of our coarse-grained models, we performed MD simulations with atomistic representations of equivalent citrate-capped AuNPs in electrolytic solutions. These simulations enabled us to calculate, at a finer resolution, the residence time of the sodium counterions. Indeed, our atomistic simulations of citrate-capped AuNPs displayed the same trend, that is, as the charge density of the NP increased, the sodium ions became more affine for the NP's surface, thus bonding for longer times (Supplementary Fig. 5).

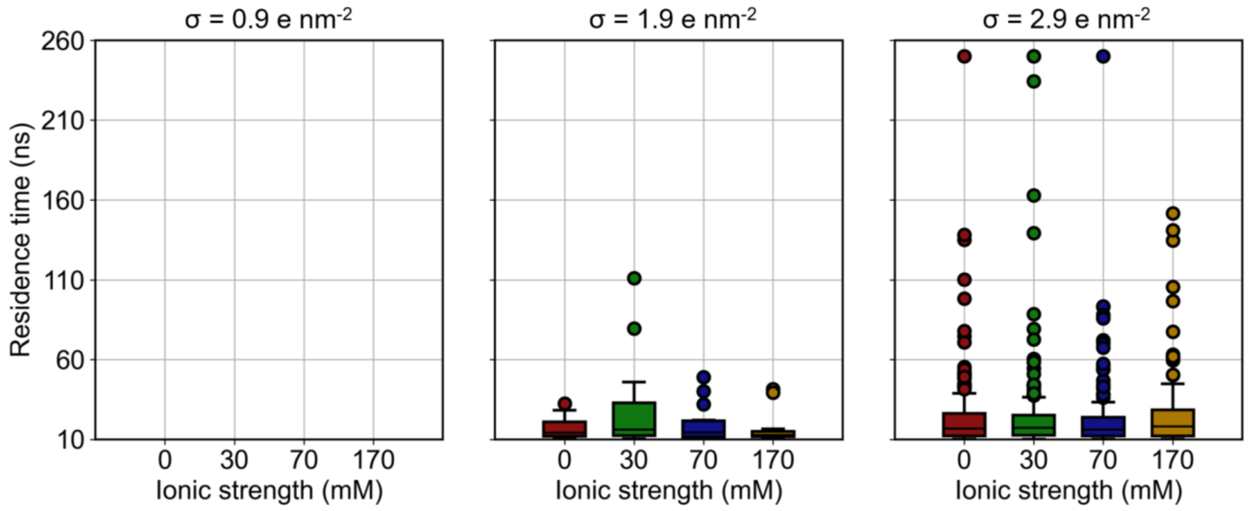

Supplementary Fig. 5 Distribution of the residence times of sodium ions onto citrate-capped AuNPs. Only the persistent binding events are considered (i.e., residence times greater than 10 ns). The boxplots show the median of the distributions, and the error bars indicate the first and third quartiles. Simulations at ionic concentrations of 0, 30, 70, and 170 mM are shown in red, green, blue, and yellow, respectively.

The setup of these atomistic simulations consisted of individual 3 nm AuNPs coated with 10, 20 and 30 monohydrogencitrate molecules, i.e., surface charge density of 0.9, 1.9, and 2.9 e nm^{-2}, respectively. The atomistic metallic core was constructed with the Wolff method implemented in the NanoCrystal web server,[8] as described in Supplementary Discussion 1, and its nonbonded parameters were taken from Heinz and co-workers.[12] The monohydrogencitrate molecules were parametrized with the GAFF force field[13] (charges derived with the RESP method[14,15]). The coating ligands, which were frozen throughout the simulation, were placed with their two terminal

carboxylate groups pointing towards the gold core, as elucidated with high-level quantum mechanical calculations.[10] The system was solvated in electrolytic solutions (I = 0, 30, 70, and 170 mM) using the TIP3P water model[16] and AMBER14SB ions.[17] Each combination of surface charge and ionic strength was simulated for 250 ns.

4. Absorbance profiles

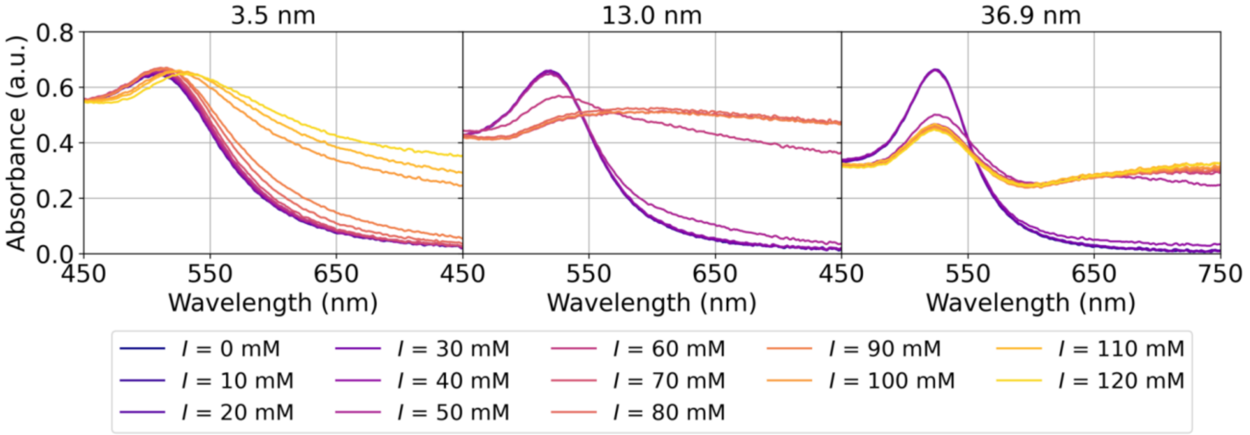

Supplementary Fig. 6 UV-Vis absorbance profiles for 3.5 (A), 13.0 (B), and 36.9 nm (C) citrate-capped AuNPs at different sodium chloride concentrations.

5. Correlation between well-depth ε and free energy of dimerization

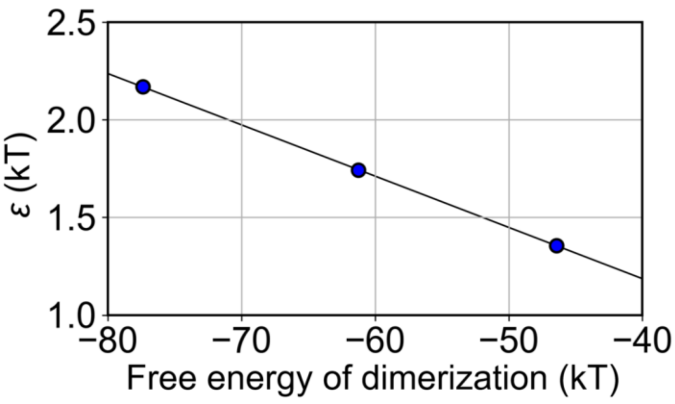

Supplementary Fig. 7 Correlation between the hydrophobicity of the metallic NPs (as described by the well-depth ε) and the free energy of NP dimerization.

6. Shear planes distributions

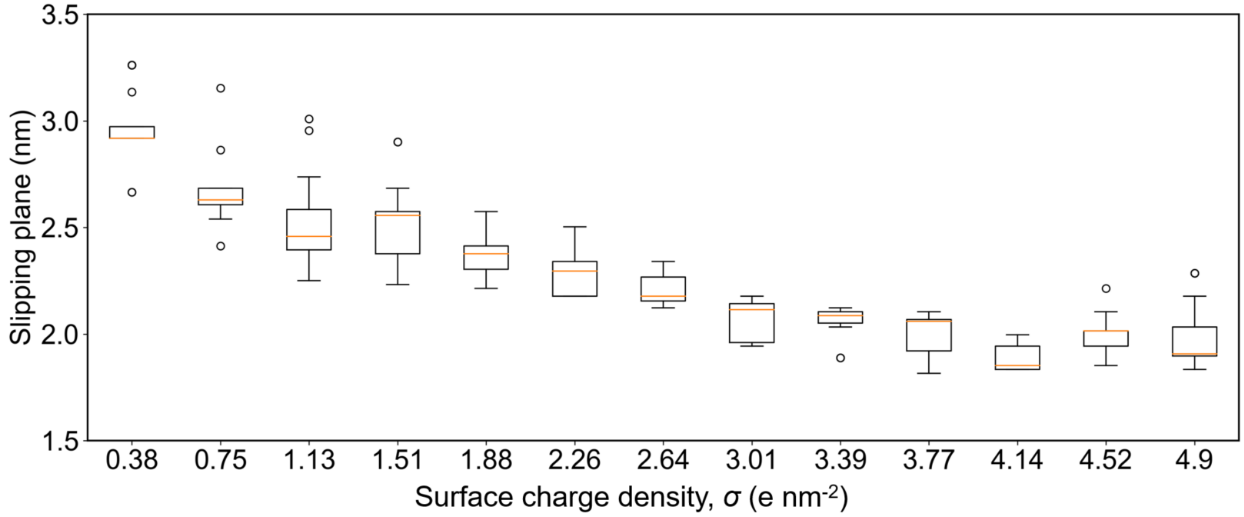

Supplementary Fig. 8 Distribution of the position of the shear planes of each surface charge density σ. The points of each distribution result from a fit for a different number of points of the sodium ions' RDFs. The boxplots show the median of the distributions, and the error bars indicate the first and third quartiles.

7. Coarse-grained models for metal NPs

a. The building of coarse-grained hydrophobic NPs

The metallic nanoparticles (NPs) employed in this study consisted of 126 beads arranged in rings stacked on top of each other as described elsewhere (Supplementary Fig. 9a).[18–21] The beads were assigned the C1 type as incorporated in the Martini v2.2 force field,[22,23] and which corresponds to a purely hydrophobic moiety. Moreover, the mass of an NP represented at the atomic level was distributed on all the available beads of the coarse-grained model. This mass corresponded to a value of 556 u.m.a per bead. The spherical shape of the NPs was retained by imposing an elastic network with a force constant of 15000 kJ mol^{-1} nm^{-2}. The elastic network united each bead with its six nearest neighbors as well as their farthest neighbor (Supplementary Fig. 9a).

b. The building of charged NPs for the building of dispersion state phase diagrams

In this study, we used implicit-ligand models for our charged metallic NPs. In detail, our capped NPs were represented as hydrophobic cores with a partial charge in certain surface beads. For the construction of these bodies, we employed the same algorithm as for pristine NPs (Supplementary Discussion 7a) modifying the charge of selected hydrophobic beads according to the target charge

of the NP (Supplementary Fig. 9b). The sites were selected by uniformly shuffling all the beads according to their spherical coordinates. The charge of each bead was chosen as -2 e to mimic the most populated deprotonation state of citrate molecules onto spherical gold NPs.[24] For example, an NP with a total charge of -40 e (charge density 1.9 e nm^{-2}), would have 20 surface beads with a charge of -2 e, each. Importantly, our charged-sphere models are valid for gold NPs coated with small, dianionic capping agents. Consequently, the phase diagrams derived from these models NPs describe the dispersion state of gold NPs in the presence of ligands like citrate, cyclic oxocarbons, and dicarboxylic acids.

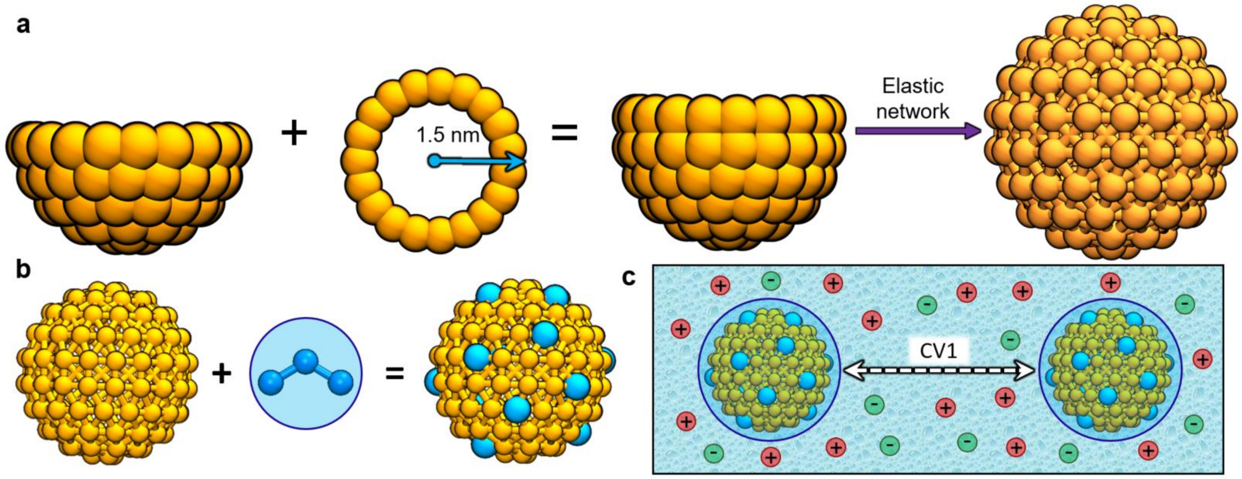

Supplementary Fig. 9 Graphical representation of the models employed for the various studied components. **a.** The pristine metallic NPs (orange) were constructed from a series of concentric rings stacked over one another and held together by an elastic network. **b.** An explicit coarse-grained model for citrate (cyan) was built as three charged beads, and it was used only to calculate ΔG^{lig}_{desolv}. The citrate-capped NPs used to derive the dispersion state phase diagrams were modeled as hollow spheres conformed by hydrophobic beads, some of which carried a formal charge. The number of charged sites was proportional to the net charge of the NP. **c.** Initial placement of two NPs immersed in a saline solution for the potential of mean force calculations. The collective variable CV1 was defined as the closest distance between the two bodies' surface.

8. AuNPs TEM images

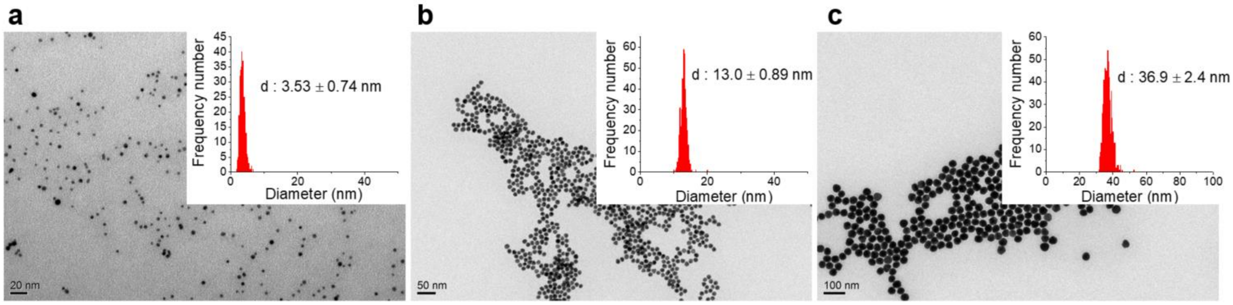

Supplementary Fig. 10 TEM images and size distribution. **a.** 3.5 nm gold nanoparticles, diameter = 3.53 ± 0.74 nm. **b.** 13.0 nm gold nanoparticles, diameter = 13.0 ± 0.89 nm. **c.** 36.9 nm gold nanoparticles, diameter = 36.9 ± 2.4 nm.

9. Supplementary references

1. Michalowsky, J., Schäfer, L. V., Holm, C. & Smiatek, J. A refined polarizable water model for the coarse-grained MARTINI force field with long-range electrostatic interactions. *J. Chem. Phys.* **146**, 054501 (2017).

2. Yesylevskyy, S. O., Schäfer, L. V., Sengupta, D. & Marrink, S. J. Polarizable water model for the coarse-grained MARTINI force field. *PLoS Comput. Biol.* **6**, 1–17 (2010).

3. Michalowsky, J., Zeman, J., Holm, C. & Smiatek, J. A polarizable MARTINI model for monovalent ions in aqueous solution. *J. Chem. Phys.* **149**, 163319 (2018).

4. Pitera, J. W. & van Gunsteren, W. F. A Comparison of Non-Bonded Scaling Approaches for Free Energy Calculations. *Mol. Simul.* **28**, 45–65 (2002).

5. Steinbrecher, T., Mobley, D. L. & Case, D. A. Nonlinear scaling schemes for Lennard-Jones interactions in free energy calculations. *J. Chem. Phys.* **127**, 214108 (2007).

6. Parrinello, M. & Rahman, A. Polymorphic transitions in single crystals: A new molecular dynamics method. *J. Appl. Phys.* **52**, 7182–7190 (1981).

7. Bennett, C. H. Efficient estimation of free energy differences from Monte Carlo data. *J. Comput. Phys.* **22**, 245–268 (1976).

8. Chatzigoulas, A., Karathanou, K., Dellis, D. & Cournia, Z. NanoCrystal: A Web-Based Crystallographic Tool for the Construction of Nanoparticles Based on Their Crystal Habit.

J. Chem. Inf. Model. **58**, 2380–2386 (2018).

9. Skriver, H. L. & Rosengaard, N. M. Surface energy and work function of elemental metals. *Phys. Rev. B* **46**, 7157–7168 (1992).

10. Al-Johani, H. *et al.* The structure and binding mode of citrate in the stabilization of gold nanoparticles. *Nat. Chem.* **9**, 890–895 (2017).

11. Wulandari, P. *et al.* Coordination of carboxylate on metal nanoparticles characterized by Fourier transform infrared spectroscopy. *Chem. Lett.* **37**, 888–889 (2008).

12. Heinz, H., Vaia, R. A., Farmer, B. L. & Naik, R. R. Accurate simulation of surfaces and interfaces of face-centered cubic metals using 12-6 and 9-6 lennard-jones potentials. *J. Phys. Chem. C* **112**, 17281–17290 (2008).

13. Wang, J., Wolf, R. M., Caldwell, J. W., Kollman, P. A. & Case, D. A. Development and testing of a general Amber force field. *J. Comput. Chem.* **25**, 1157–1174 (2004).

14. Vanquelef, E. *et al.* R.E.D. Server: A web service for deriving RESP and ESP charges and building force field libraries for new molecules and molecular fragments. *Nucleic Acids Res.* **39**, W511–W517 (2011).

15. Wang, J., Cieplak, P. & Kollman, P. A. How Well Does a Restrained Electrostatic Potential (RESP) Model Perform in Calculating Conformational Energies of Organic and Biological Molecules? *J. Comput. Chem.* **21**, 1049–1074 (2000).

16. Jorgensen, W. L., Chandrasekhar, J., Madura, J. D., Impey, R. W. & Klein, M. L. Comparison of simple potential functions for simulating liquid water. *J. Chem. Phys.* **79**, 926–935 (1983).

17. Chen, A. A. & Pappu, R. V. Parameters of monovalent ions in the AMBER-99 forcefield: Assessment of inaccuracies and proposed improvements. *J. Phys. Chem. B* **111**, 11884–11887 (2007).

18. Gkeka, P., Sarkisov, L. & Angelikopoulos, P. Homogeneous hydrophobic-hydrophilic surface patterns enhance permeation of nanoparticles through lipid membranes. *J. Phys. Chem. Lett.* **4**, 1907–1912 (2013).

19. Angelikopoulos, P., Sarkisov, L., Cournia, Z. & Gkeka, P. Self-assembly of anionic, ligand-coated nanoparticles in lipid membranes. *Nanoscale* **9**, 1040–1048 (2017).

20. Gkeka, P. & Angelikopoulos, P. The Role of Patterned Hydrophilic Domains in Nanoparticle-Membrane Interactions. *Curr. Nanosci.* **7**, 690–698 (2011).

21. Gkeka, P., Angelikopoulos, P., Sarkisov, L. & Cournia, Z. Membrane Partitioning of Anionic, Ligand-Coated Nanoparticles Is Accompanied by Ligand Snorkeling, Local Disordering, and Cholesterol Depletion. *PLoS Comput. Biol.* **10**, e1003917 (2014).

22. Marrink, S. J., Risselada, H. J., Yefimov, S., Tieleman, D. P. & De Vries, A. H. The MARTINI force field: Coarse grained model for biomolecular simulations. *J. Phys. Chem. B* **111**, 7812–7824 (2007).

23. De Jong, D. H. *et al.* Improved parameters for the martini coarse-grained protein force field. *J. Chem. Theory Comput.* **9**, 687–697 (2013).

24. Park, J. W. & Shumaker-Parry, J. S. Structural study of citrate layers on gold nanoparticles: Role of intermolecular interactions in stabilizing nanoparticles. *J. Am. Chem. Soc.* **136**, 1907–1921 (2014).
